# A composite light-harvesting layer from photoactive polymer and halide perovskite for planar heterojunction solar cells

**DOI:** 10.1038/srep29567

**Published:** 2016-07-14

**Authors:** Heming Wang, Yaqub Rahaq, Vikas Kumar

**Affiliations:** 1 Materials & Engineering Research Institute, Sheffield Hallam University, City Campus, Howard Street, Sheffield, S1 1WB, UK.

## Abstract

A new route for fabrication of photoactive materials in organic-inorganic hybrid solar cells is presented in this report. Photoactive materials by blending a semiconductive conjugated polymer with an organolead halide perovskite were fabricated for the first time. The composite active layer was then used to make planar heterojunction solar cells with the PCBM film as the electron-acceptor. Photovoltaic performance of solar cells was investigated by J-V curves and external quantum efficiency spectra. We demonstrated that the incorporation of the conjugated photoactive polymer into organolead halide perovskites did not only contribute to the generation of charges, but also enhance stability of solar cells by providing a barrier protection to halide perovskites. It is expected that versatile of conjugated semi-conductive polymers and halide perovskites in photoactive properties enables to create various combinations, forming composites with advantages offered by both types of photoactive materials.

Polymer solar cells (PSCs) have received considerable investigations in the past twenty years owing to their potentially low-cost roll-to-roll processing methods. The power conversion efficiency (PCE) has steadily increased to ~10% for a single bulk heterojunction (BHJ) photovoltaic (PV) device^1^,^2^. However, the PCE is still too low to occupy a large market share when competed with 1^st^ generation Si-wafer-based and 2^nd^ generation thin-film-based solar cells. The low PCE is mainly due to their short charge-carrier diffusion length within the photoactive conjugated polymer^3^,^4^,^5^, which causes the recombination of positive and negative charges. Because of short charge-carrier diffusion length, planar heterojuncntion (PHJ) architectures only result in inefficient PSCs. High-efficiency is achieved in BHJ PSCs via the blend of a photoactive polymer with an electron-acceptor (e.g. phenyl-C61-butyric acid methyl ester (PCBM) or phenyl C71 butyric acid methyl ester (PC_71_BM))^6^. In addition, organolead halide perovskite-based solar cells more recently demonstrated high-efficient capability of converting sunlight into electricity with low cost precursors and cheap solution-processing methods. The PCE has significantly increased from 3.8% in 2009^7^ to ~21% up to date^8^. The rapid increase in PCE has brought remarkable rise in interest in this fascinating class of materials. By combining different halides and altering their ratios in organolead halide perovskites, light absorbance can be tuned to cover a wide range of wavelengths^9^. However, their poor stability remains one of the main issues except containing toxic lead for the large-scale commercial deployment. Long term stability is affected by many effects including external factors of moisture, oxygen, temperature, UV light and internal intrinsic factors of ion migration, electro-migrations and interfacial reactions^10^. In terms of the PV devices, stability can be related to device architectures, electrodes, interfacial layers (hole- and electron-blocking layer), and active layers of the halide perovskites. Studies are widely carried out in these aspects and significant improvements have been achieved; e.g. Organic-inorganic hybrid combination of Cs and formamidinium iodide (FAI) as A in ABX_3_ perovskite structures demonstrated significant enhancement in thermal stability of organolead halide perovskites^11^,^12^. Moreover, by incorporating polyethylene glycol into the methyl ammonium lead iodide (MAPbI_3_) perovskite active layer has not only increased its moisture resistance but also showed a “self-healing” effect on degradation of the PCE^13^.

In this work, we are the first to report a fabrication method of organic-inorganic hybrid photoactive layers by blending a conjugated photoactive polymer with a halide perovskite to form a composite active material for light-harvesting in solar cells. The blend forms PHJ architectural solar cells with a PCBM layer, which can perform a high-efficient PCE. This approach illustrates a new route to develop organic-inorganic hybrid solar cells. Photoactive conjugated polymers include a family of organic semiconductive materials that covers a full range of wavelengths for light absorption; e.g. poly[3-hexylthiophene-2,5-diyl] (P3HT) with ~2.0 eV band gap, poly[[4,8-bis[(2-ethylhexyl)oxy]benzo[1,2-b:4,5-b′]dithiophene-2,6-diyl][3-fluoro-2-[(2-ethylhexyl)carbonyl]thieno[3,4-b]thiophenediyl]] (PTB-7) with ~1.81 eV band gap, and poly[diketopyrrolopyrrole-terthiophene] (PDPP3T) with ~1.56 eV band gap. This will allow more flexibility or better opportunity in synthesizing a photoactive layer to extend its light absorbance into full-range effective wavelengths without sacrificing other properties such as stability of organometal halide perovskites; e.g. Organometal bromine-based perovskite has much better stability than iodide-based perovskite but suffers narrow range wavelengths in light absorbance^14^,^15^,^16^. The conjugated polymer can also provide additional properties such as barrier protection to halide perovskites for stability enhancement of solar cells since the conjugated semiconductive polymers have better moisture resistance than organometal halide perovskites.

## Results

**Figure 1:**
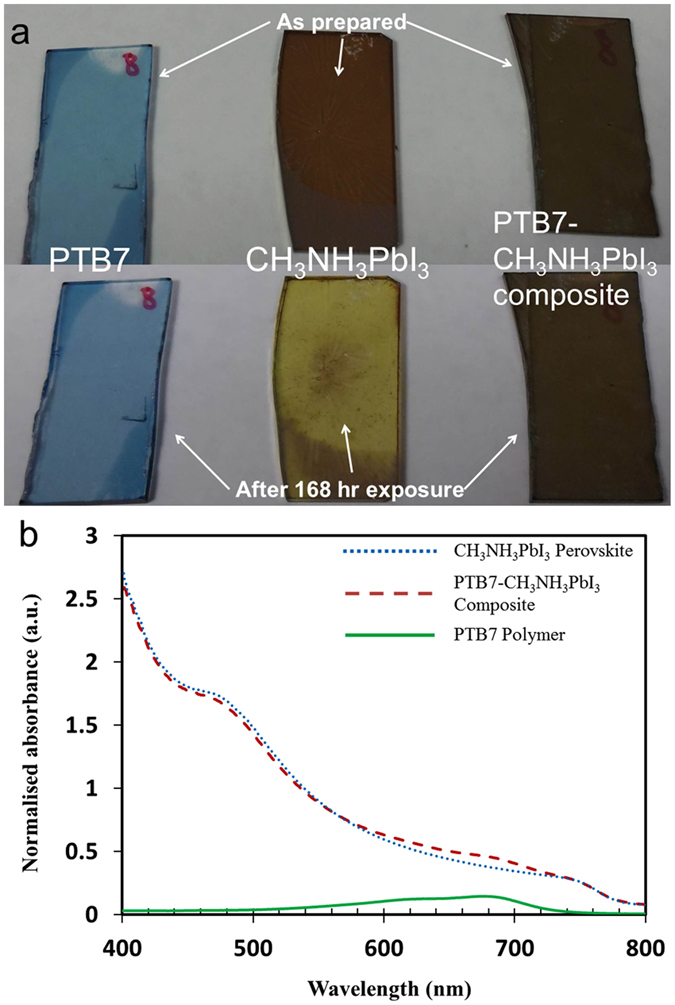
Photos of active layers at ambient environment with ~35% humidity and light absorbance after preparation: (**a**) Top: PTB7, CH_3_NH_3_PbI_3_, and PTB7-CH_3_NH_3_PbI_3_ composite after preparation; Bottom: PTB7, CH_3_NH_3_PbI_3_, and PTB7-CH_3_NH_3_PbI_3_ composite after 168 hours exposure; (**b**) Absorption spectra.

**Figure 2:**
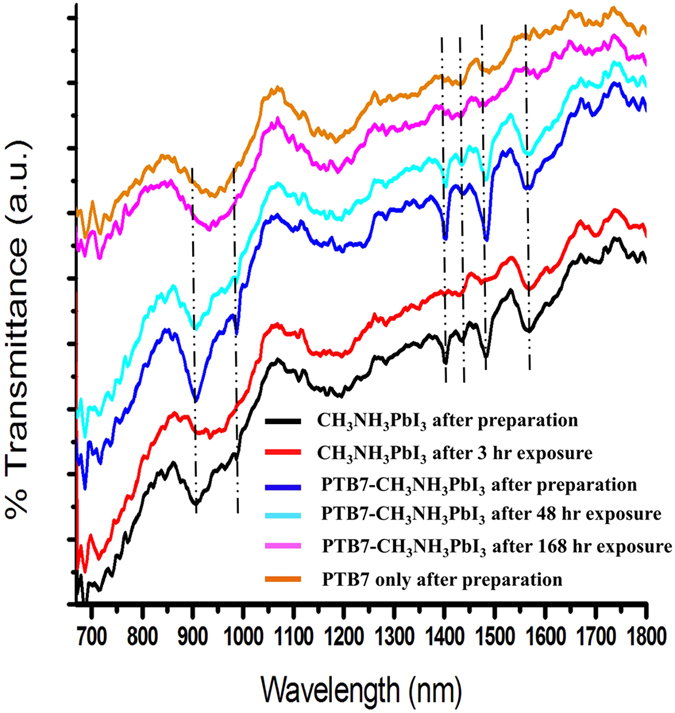
FTIR spectra of photoactive thin films at various conditions.

There are many potential combinations that could be designed or selected to create a composite photoactive layer from various semiconductive conjugated polymers and halide perovskites for solar cells. To demonstrate the formation of this novel composite active layer, we used an example: PTB7 photoactive polymer and CH_3_NH_3_PbI_3_ organolead perovskite for fabrication of our composite active layer. PbI_2_ and PTB7 thin films were previously deposited on glass substrates by the spin-coating method and the methylammonium iodide (MAI) solution was then added on top of the pre-deposited film. After completion of the chemical reaction between PbI_2_ and MAI, the PTB7-CH_3_NH_3_PbI_3_ composite thin film was formed by the spin-coating method and thereafter was heated at 100 °C on a hotplate in the glovebox. For comparative studies, the two-step method was also utilized to produce the pristine CH_3_NH_3_PbI_3_ perovskite thin film; i.e. deposition of the PbI_2_ thin film and then formation of the CH_3_NH_3_PbI_3_ film by applying the MAI solution on top of the PbI_2_ film. Figure 1 presents photos of thin films and light absorbance of PTB7, CH_3_NH_3_PbI_3_, and PTB7-CH_3_NH_3_PbI_3_ composite in the wavelength range of 400 to 800 nm on glass substrates, respectively. Tremendous enhancement in stability of CH_3_NH_3_PbI_3_ perovskite due to forming the PTB7-CH_3_NH_3_PbI_3_ composite material was verified after samples of PTB7, CH_3_NH_3_PbI_3_, and PTB7-CH_3_NH_3_PbI_3_ composite were left at ambient environment with ~35% humidity. As shown in Fig. 1a, initial dark-brown color of the pristine CH_3_NH_3_PbI_3_ perovskite changed to yellow color after 168 hours exposure because of decomposition of CH_3_NH_3_PbI_3_ into PbI_2_^17^ while unaltered dark-brown color of the PTB7-CH_3_NH_3_PbI_3_ composite film appears. The PTB7 polymer absorbs visible light mainly in the wavelength range of 550 to 750 nm in Fig. 1b. Contribution to harvesting of solar radiation via the incorporation of PTB7 into the CH_3_NH_3_PbI_3_ perovskite film is evidenced in this wavelength range when compared with absorbance of the pristine CH_3_NH_3_PbI_3_ film. The emphasized light absorption spectra of PTB7, CH_3_NH_3_PbI_3_, and PTB7-CH_3_NH_3_PbI_3_ composite are illustrated in Fig. S1. A slightly lower light absorption for the PTB7-CH_3_NH_3_PbI_3_ composite film against the pristine CH_3_NH_3_PbI_3_ film is observed in the wavelength range of 400 to 550 nm in Fig. 1b, which is attributed to slightly smaller amount of CH_3_NH_3_PbI_3_ by comparing the same thickness of photoactive layers with each other. We recorded ATR-FTIR spectra of photoactive thin films as shown in Fig. 2. After preparation, CH_3_NH_3_PbI_3_ perovskite and PTB7-CH_3_NH_3_PbI_3_ composite thin films illustrate main characteristic peaks of lead perovskite crystals; i.e. wide strong peak at ~910 cm^−1^ for CH_3_-NH_3_ rock, peak at ~989 cm^−1^ for C-N stretch, peaks at ~1403 cm^−1^ and ~1439 cm^−1^ for C-H vibration bands, and peaks at ~1482 cm^−1^ and ~1570 cm^−1^ for H-N vibration bands^17^. Most vibrational peaks for the pure CH_3_NH_3_PbI_3_ perovskite either disappeared or became weak after exposure for 3 hr under ambient air although the dark color of the film observed no change. On contrast, the PTB7-CH_3_NH_3_PbI_3_ composite thin film maintained its vibrational peaks with only slightly reduced intensity after 48 hr exposure to ambient environment. Its vibrational peaks disappeared after 168 hr exposure under ambient air, presenting the same FTIR spectrum as that of the pure PTB7 film. However, no changed color was revealed as previously shown in Fig. 1a.

**Figure 3:**
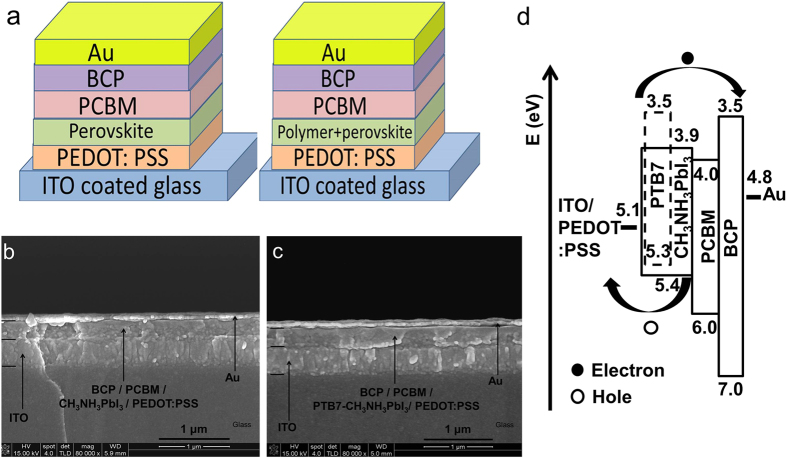
Device architecture and operational mechanism: (**a**) schematic architecture diagram of the PV devices fabricated from CH_3_NH_3_PbI_3_ and PTB7-CH_3_NH_3_PbI_3_ composite, respectively; (**b**) Cross-section SEM images of real PV devices; (**c**) Energy level schematic diagram of the PV device from PTB7-CH_3_NH_3_PbI_3_ composite. Similar diagram for CH_3_NH_3_PbI_3_ based PV devices can be drawn.

**Figure 4:**
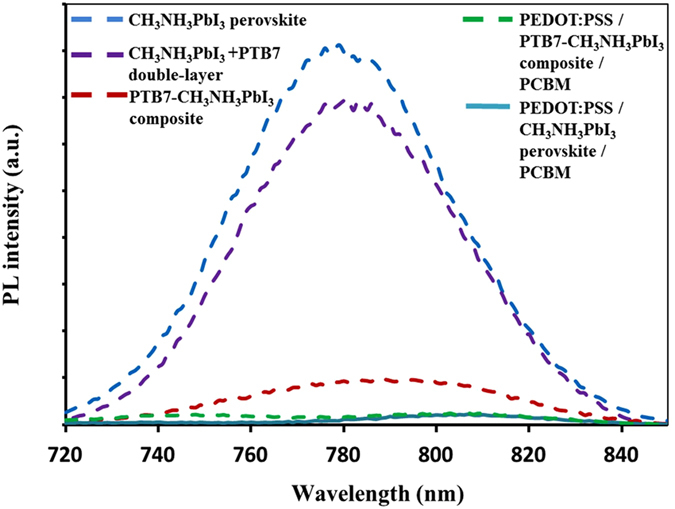
Emission peaks in steady-state photoluminesence spectra upon excitation 400 nm under the same experimental conditions with the same size of samples on glass substrates.

We separately used the pristine CH_3_NH_3_PbI_3_ perovskite and PTB7-CH_3_NH_3_PbI_3_ composite films as photoactive layers to fabricate our PV devices, enabling to understand influences of the PTB7 photoactive polymer on PCE after it was introduced into CH_3_NH_3_PbI_3_ perovskite. Figure 3 presents the schematic device architecture of solar cells combining cross-sectional scanning electron microscope (SEM) images of the integrated devices and their operational mechanism. The devices were constructed based on the planar-type architecture in Fig. 3a with a structure of indium tin oxide (ITO)/poly(3,4-ethylenedioxythiophene) polystyrene sulfonate (PEDOT:PSS)/ CH_3_NH_3_PbI_3_ perovskite or PTB7-CH_3_NH_3_PbI_3_ polymer-perovskite composite/ PCBM/bathocuproine (BCP)/Au, where the PEDOT:PSS and BCP layers act as the hole collecting and buffer layers respectively. Unfortunately SEM cross section images cannot provide us contrast to differentiate 4 layers of BCP, PCBM, CH_3_NH_3_PbI_3_ or PTB7-CH_3_NH_3_PbI_3_, and PEDOT:PSS in Fig. 3b,c. The estimated thicknesses of BCP, PCBM, and PEDOT:PSS are ~10 nm, ~50 nm, and ~30 nm, separately. However, this combined four-layer in Fig. 3b,c reveals the same thickness of ~300 nm for CH_3_NH_3_PbI_3_ or PTB7-CH_3_NH_3_PbI_3_ based solar cells, inferring that the photoactive layer of CH_3_NH_3_PbI_3_ perovskite or PTB7-CH_3_NH_3_PbI_3_ composite presents the same thicknesses in both PV devices. Cross section SEM images of single layer, CH_3_NH_3_PbI_3_ perovskite or PTB7-CH_3_NH_3_PbI_3_ composite on the Si substrate, further confirm that the thickness of the photoactive layer is ~210 nm in Fig. S2. Figure 3d illustrates the operation mode of the PTB7-CH_3_NH_3_PbI_3_ composite-based PV device and the energy levels of each component layer in the device. PTB7 and CH_3_NH_3_PbI_3_ absorb the UV–vis solar radiation and generate excitons, which can be dissociated into free holes and electrons at the interfaces of PTB7/CH_3_NH_3_PbI_3_, PEDOT:PSS/CH_3_NH_3_PbI_3_, and CH_3_NH_3_PbI_3_/PCBM, respectively. The BCP layer is used as a buffer layer to form good contact with the Au electrode for electrons collection. Holes generated by PTB7 and CH_3_NH_3_PbI_3_ are efficiently collected at the anode due to high hole mobility of perovskite and negligible energy level difference between the highest occupied molecular orbital of PTB7 and the valence band of CH_3_NH_3_PbI_3_ perovskite. We investigated steady-state photoluminescence spectra of thin films for CH_3_NH_3_PbI_3_ perovskite, PTB7-CH_3_NH_3_PbI_3_ composite, and CH_3_NH_3_PbI_3_ + PTB7 double-layer (i.e. a PTB7 thin film on top of the pristine CH_3_NH_3_PbI_3_ film) under the same experimental conditions with the same size of samples on glass substrates. Emission peaks upon excitation 400 nm in photoluminescence spectra for CH_3_NH_3_PbI_3_, PTB7-CH_3_NH_3_PbI_3_ composite, and CH_3_NH_3_PbI_3_ + PTB7 double-layer are shown separately in Fig. 4. Thin films of the pristine CH_3_NH_3_PbI_3_ and CH_3_NH_3_PbI_3_ + PTB7 double-layers illustrate very close intensity for the photoluminescence peak at ~780 nm. Compared to thin films of CH_3_NH_3_PbI_3_ and CH_3_NH_3_PbI_3_ + PTB7 double-layer, the PTB7-CH_3_NH_3_PbI_3_ composite film illustrates clear quench effect, confirming the effective charge transportation between PTB7 and CH_3_NH_3_PbI_3_ within the blend. Steady-state photoluminescence spectra of triple layers of PEDOT:PSS, CH_3_NH_3_PbI_3_ perovskite or PTB7-CH_3_NH_3_PbI_3_ composite, and PCBM are also presented in Fig. 4, respectively. Again, significant quench effects were observed. Photoluminescence peaks at ~780 nm nearly disappear after forming the planar-type architecture with PEDOT:PSS and PCBM. The same results in photoluminescence spectra were achieved in Fig. S3 upon excitation 620 nm except smaller percentage of the quenching effect in comparative with those upon excitation 400 nm.

**Figure 5:**
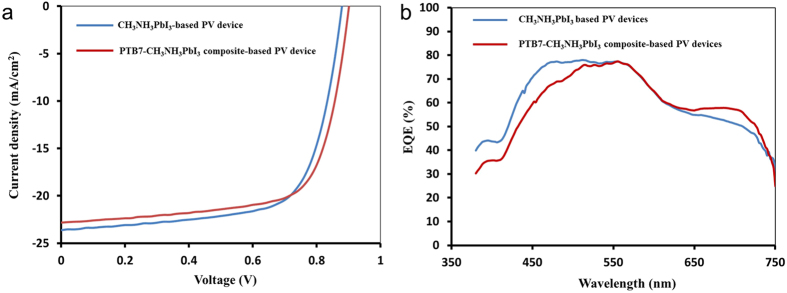
Performance of solar cells: (**a**) J-V curves of devices from CH_3_NH_3_PbI_3_ and PTB7-CH_3_NH_3_PbI_3_ composite; (**b**) EQE of devices from CH_3_NH_3_PbI_3_ and PTB7-CH_3_NH_3_PbI_3_ composite, respectively.

We characterized performance of our solar cells based on CH_3_NH_3_PbI_3_ or PTB7-CH_3_NH_3_PbI_3_ composite photoactive layers by J-V curves and external quantum efficiency (EQE) spectra. The best J-V performances are separately shown in Fig. 5a. The mean for PCE of the PTB7-CH_3_NH_3_PbI_3_ blend-based solar cells (the highest PCE = 14.4%) is 12.51% with standard deviation = 0.99 and the means of open circuit voltage (V_oc_), current density (J_sc_), and fill factor (FF) are 0.88 V, 22.28 mA/cm^2^, and 63% with standard deviations of 0.031, 0.76, and 0.027 respectively shown in Table S1. The mean for PCE of CH_3_NH_3_PbI_3_ based solar cells (the highest PCE = 14.3%) is 12.76% with standard deviation = 1.01 and the means of V_oc_, J_sc_ and FF are 0.86 V, 20.88 mA/cm^2^, and 70% with standard deviations of 0.015, 1.65, and 0.019 separately presented in Table S2. The incorporation of PTB7 polymer into CH_3_NH_3_PbI_3_ perovskite results in higher V_oc_, more uniformly distributed higher J_sc_ but slightly lower FF than those of the CH_3_NH_3_PbI_3_ based devices when an equivalent thickness of the photoactive layer was fabricated in the solar cells, implying that charge transport can efficiently extract in the composite photoactive layer. Since light absorbance of PTB7 mostly overlaps with CH_3_NH_3_PbI_3_ in the wavelength range of ~550 to 750 nm, both solar cells present a close PCE. EQE spectra may provide addition information of PTB7’s contribution on generating charges and current output as shown in Fig. 5b. One can clearly observe their differences between CH_3_NH_3_PbI_3_ and PTB7-CH_3_NH_3_PbI_3_ based solar cells in EQE spectra. The CH_3_NH_3_PbI_3_ based solar cells illustrate higher EQE in the wavelength range of ~380 to 550 nm and more level curve in the wavelength range from ~480 to 550 nm than those of the PTB7-CH_3_NH_3_PbI_3_ based solar cells. This is probably caused by alternation of microstructures and components in the photoactive materials due to the addition of PTB7 into the pristine CH_3_NH_3_PbI_3_ when the same thickness of a photoactive layer was utilized in the solar cells. Inversely, higher EQE is achieved for the PTB7-CH_3_NH_3_PbI_3_ based solar cells in the wavelength range of ~610 to 750 nm and more level curve is presented in the wavelength range from ~650 to 700 nm. Moreover, the CH_3_NH_3_PbI_3_ based solar cells have hysteresis-less behavior in Fig. S4a, indicating good quality of the perovskite films and efficient charge extraction throughout the devices. Compared to the CH_3_NH_3_PbI_3_ based PV devices, the PTB7-CH_3_NH_3_PbI_3_ based PV devices illustrate tiny difference in the J-V curves between the forward scan and reverse scan at the scan rate of 0.01 V/s in Fig. S4b. Different hysteresis behaviour between CH_3_NH_3_PbI_3_ and PTB7-CH_3_NH_3_PbI_3_ based solar cells further evidences that the addition of the conjugated PTB7 polymer into CH_3_NH_3_PbI_3_ perovskite may slightly change its property on charge extraction.

**Figure 6:**
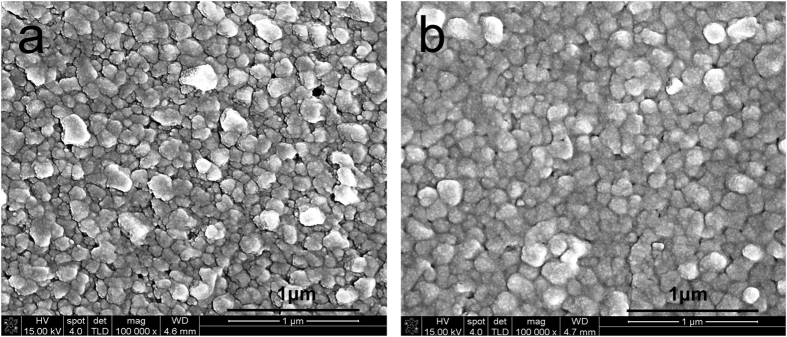
SEM morphologies of photoactive thin films: (**a**) CH_3_NH_3_PbI_3_ perovskite; (**b**) PTB7-CH_3_NH_3_PbI_3_ composite.

**Figure 7:**
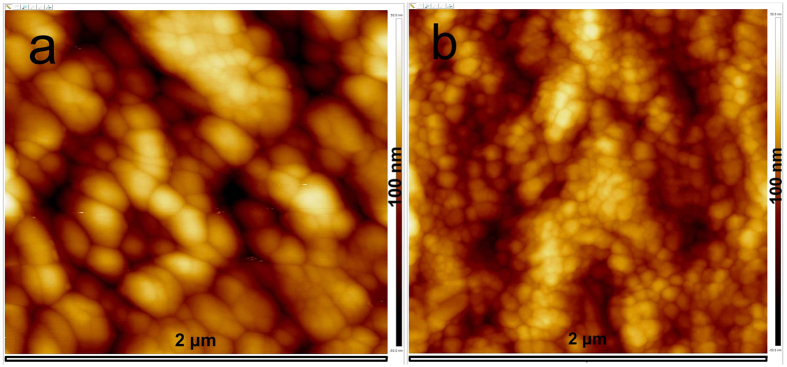
AFM morphologies of photoactive thin films: (**a**) CH_3_NH_3_PbI_3_ perovskite; (**b**) PTB7-CH_3_NH_3_PbI_3_ composite. Scale bars are 2 × 2 μm and height bars are 100 nm.

We then performed structural characterization of the photoactive layers. XRD pattern verified their perovskite structures for both thin films of CH_3_NH_3_PbI_3_ and PTB7-CH_3_NH_3_PbI_3_ composite as presented in Fig. S5. Morphologies of CH_3_NH_3_PbI_3_ and PTB7-CH_3_NH_3_PbI_3_ composite thin films were respectively investigated by SEM and atomic force microscope (AFM). Both thin films of CH_3_NH_3_PbI_3_ perovskite and PTB7-CH_3_NH_3_PbI_3_ composite illustrate morphologies with polycrystalline grains (perovskite phase) in Fig. 6. However, tiny gaps between some grains in the pristine CH_3_NH_3_PbI_3_ film are observed in Fig. 6a while fully connected grain phases appeared in the PTB7-CH_3_NH_3_PbI_3_ composite film in Fig. 6b. We also noticed that image contrast was reduced for the PTB7-CH_3_NH_3_PbI_3_ composite film due to the addition of PTB7 into CH_3_NH_3_PbI_3_ when compared with SEM images of the pristine CH_3_NH_3_PbI_3_ film under the same operational parameters. Therefore, the investigation of morphologies by SEM inferred that the PTB7 polymer was blended into CH_3_NH_3_PbI_3_ perovskite, forming nanocomposites with denser structures. AFM images are presented both in Fig. 7 and Fig. S6, wherein different morphological structures were also revealed between CH_3_NH_3_PbI_3_ perovskite and PTB7-CH_3_NH_3_PbI_3_ composite thin films. The CH_3_NH_3_PbI_3_ perovskite film has grains with the size of larger than 100 nm in Fig. 7a while the size of grains in the PTB7-CH_3_NH_3_PbI_3_ composite film is less than 50 nm in Fig. 7b. Nevertheless, as observed in SEM images, grain sizes in the pristine CH_3_NH_3_PbI_3_ and PTB7-CH_3_NH_3_PbI_3_ composite films are very close at more than 100 nm. Therefore, we assign that much smaller grain in AFM images for the PTB7-CH_3_NH_3_PbI_3_ composite film was probably caused by the PTB7 polymer. Because organic materials with light elements are difficult to obtain their direct high-contrast nano-structural images by SEM^18^, only larger grains of perovskites clearly appeared in morphologies in Fig. 6. This may explain their differences on grain sizes by SEM and AFM separately.

**Figure 8:**
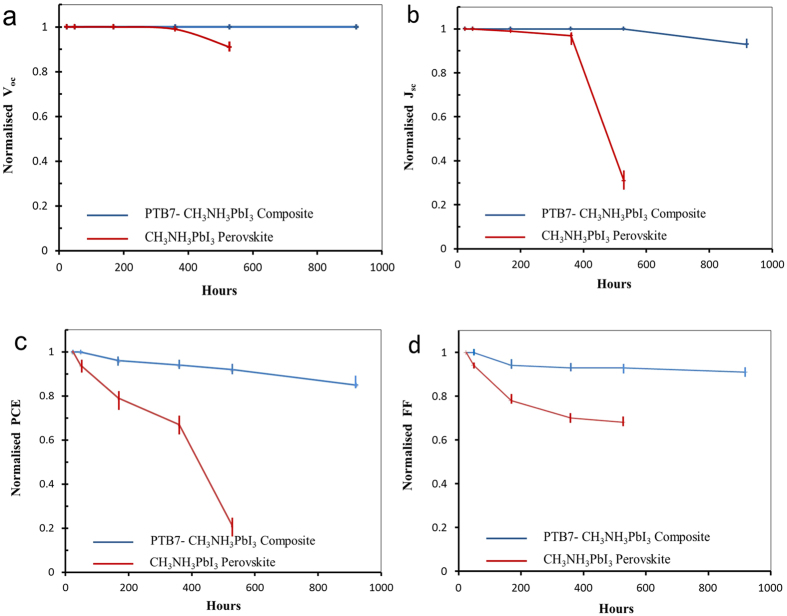
Performance variation of solar cells stored in the glovebox against time: (**a**) V_oc_ variation; (**b**) J_sc_ variation; (**c**) PCE variation; (**d**) FF variation.

We then kept our solar cells in the glovebox to investigate their stability under dark conditions except performing the J-V characterization at ambient environment. The results are presented in Fig. 8a–d. The PTB7-CH_3_NH_3_PbI_3_ based solar cells show significantly enhanced stability than the CH_3_NH_3_PbI_3_ based solar cells since the PTB7-CH_3_NH_3_PbI_3_ composite film illustrated a greatly increased resistance against decomposition of the CH_3_NH_3_PbI_3_ perovskite as previously shown in Fig. 1a. V_oc_ of the PTB7-CH_3_NH_3_PbI_3_ based solar cells maintained its original value after 920-hour-storage while V_oc_ of the CH_3_NH_3_PbI_3_ based solar cells started reducing after 360-hour-storage and dropped to ~90% of its original value only after 528-hour-storage in Fig. 8a. Simultaneously, J_sc_ of the PTB7-CH_3_NH_3_PbI_3_ based solar cells presented no change before 528-hour-storage and ~93% of its original value even after 920-hour-storage in Fig. 8b. On contrast, J_sc_ of the CH_3_NH_3_PbI_3_ based solar cells showed a noticeable decrease after 360-hour-storage while a sharply reduced value was measured at ~31% of its original value only after 528-hour-storage. Spontaneously, changes in PCE were recorded in Fig. 8c. The PCE of the PTB7-CH_3_NH_3_PbI_3_ based solar cells kept ~92% and ~85% of its original value respectively after 528- and 920-hour-storage. However, the PCE of the CH_3_NH_3_PbI_3_ based solar cells decreased to ~79% and ~21% of its original value separately only after 168- and 528-hour-storage. Variations of FF were presented in Fig. 8d. FF of the PTB7-CH_3_NH_3_PbI_3_ based solar cells kept within more than 90% of its original value in the storage period while a gradually reduced FF of the CH_3_NH_3_PbI_3_ based solar cells was illustrated, revealing only ~47% of its original value after 528-hour-storage. Therefore, one of the main factors on degrading performance of our solar cells is owing to the decrease of J_sc_ that could be caused by alternation or decomposition of CH_3_NH_3_PbI_3_ perovskite in the photoactive layer^19^. Performance degradation in ambient air with ~35% humidity for our solar cells were also recorded as shown in Fig. S7a–d. Both devices kept nearly no change for V_oc_ in Fig. S7a after 172-hour-exposure. J_sc_, PCE, and FF of both devices all gradually reduced with exposure time. However, the PTB7-CH_3_NH_3_PbI_3_ based solar cells maintained ~68% J_sc_, 64% PCE, and 75% FF of their original values after 172-hour-exposure while the CH_3_NH_3_PbI_3_ based solar cells only kept ~37% J_sc_, 18% PCE, and 43% FF of their original values.

## Discussion

FTIR spectra illustrate that vibration bands in the NH_3_CH_3_ organic groups in iodide lead perovskite structures were significantly enhanced by forming nanocomposites with the photoactive polymer PTB7. Further observation of morphologies by both SEM and AFM images suggest that the PTB7 polymer likely stayed on surfaces of CH_3_NH_3_PbI_3_ grains, which creates a barrier layer for the protection of CH_3_NH_3_PbI_3_ perovskite. Therefore, the incorporation of the photoactive PTB7 polymer hinders decomposition of lead perovskite and enhances the stability of solar cells. The weight ratio of PTB7:CH_3_NH_3_PbI_3_ in the current work is estimated at 1:80. The concentration of PTB7 in the PTB7-CH_3_NH_3_PbI_3_ composite can be increased and therefore there is a great potential to further improve the stability of the PTB7-CH_3_NH_3_PbI_3_ composite-based solar cells.

## Conclusion

We demonstrated to fabricate organic-inorganic hybrid solution-processed solar cells using blend of PTB7 polymer and CH_3_NH_3_PbI_3_ perovskite as the light-absorbing layer. The PHJ PV devices fabricated from CH_3_NH_3_PbI_3_ perovskite or PTB7-CH_3_NH_3_PbI_3_ composite with PCBM illustrated the same level of PCE. However, uniformly distributed high J_sc_ in PV devices and significant improvement in stability against decomposition of CH_3_NH_3_PbI_3_ perovskite were achieved in the PTB7-CH_3_NH_3_PbI_3_ composite, leading to the stability enhancement of solar cells. Considering the existence of a variety of family materials either for photoactive conjugated polymers or organometal halide perovskites, various combinations are anticipated for exploring high performance and low-cost organic-inorganic hybrid solar cells.

## Experimental Section

### Materials

MAI was synthesised via the chemical reaction of 27 ml methylamine solution (CH_3_NH_2_, 40 wt.% in methanol, TCI) with 30 ml of hydriodic acid (HI 57 wt.% in water, Aldrich) in an ice bath for 2 h. The methylamine solution was added first into the round-bottom flask and then HI was dropwise added in during stirring. MAI precipitates were collected after the solution was transformed into a rotary evaporator and heated at 50 °C for 1 h. The white precipitates were washed three times with diethyl ether and finally dried in vacuum for 24 h. The PbI_2_ solution was prepared by dissolving 1 mole PbI_2_ in 1 ml DMF solvent and stirred at 70 °C and then 20 μl of DIO was added into the solution to promote the dissolution of PbI_2_. The PbI_2_ solution became clear after continuously stirring at 70 °C for overnight. Thereafter, 1.0 wt.% MAI solution was then produced by adding MAI in 2-propanol and stirred for 10 min at 50 °C. The PTB7 and PCBM solution were respectively prepared by dissolving 4 mg and 30 mg of PCBM in 1 ml of chlorobenzene. 2 mg of BCP was dissolved in 1 ml of methanol to form the BCP solution. The Al4083 PEDOT:PSS solution was used as received from Ossila (1.3 to 1.7 wt.% water solution).

### Fabrication of the PV devices

Pro-structural ITO-coated glass substrates (sheet resistance 15 Ω/□) with size of ~20 × 25 mm were cleaned by soap water and then washed by DI water. After N_2_ blowing dry, the samples were ultrasonically cleaned in acetone and 2-propanol separately and then followed dry by N_2_ blow. The cleaned ITO substrates were spin-coated at 3000 rpm by the PEDOT:PSS solution and followed by heating at 140 °C for 10 min. The PbI_2_ thin film was prepared on top of the PEDOT:PSS film by the spin-coating method at 5000 rpm using the supersaturated PbI_2_ DMF solution and then annealed at 70 °C for 8 min on the hotplate. Thereafter, the PTB7 film was deposited by the spin-coating method on top of the PbI_2_ film and annealed for 2 min at 70 °C for the PTB7-CH_3_NH_3_PbI_3_ based solar cells. The MAI solution was then added either on top of the PbI2 or PTB7 film for CH_3_NH_3_PbI_3_ or PTB7-CH_3_NH_3_PbI_3_ based solar cells respectively and was kept at 1 or 2.5 min separately for the chemical reaction between PbI_2_ and MAI, followed a rotation at 4000 rpm using the spin-coater. The obtained thin films were finally heated at 100 °C for 1 h in the glovebox. The weight ratio of PTB7 to CH_3_NH_3_PbI_3_ in the fabricated composite layer was estimated as ~1:80. The PCBM film was then deposited by the spin-coating method from the PCBM solution on top of the formed active layer at 2000 rpm and followed heated at 100 °C for 30 min. Thereafter, the thin film of BCP about 10-nm-thick was fabricated by the spin-coating method on top of the BCPM thin film as the buffer layer and finally the Au thin film as the back contact was applied on top of the BCP film by the vacuum sputtering method. The Au thin film was about 100 nm.

### Characterisation

J-V curves were performed under the simulated AM 1.5G irradiation (100 mW /cm^2^) using keithley 2401 sourcemeter in ambient environment. A RR267MON Si-based solar cell was utilised to calibrate light intensity of the solar light simulator with the mismatch factor one before J-V measurement were carried out. An aperture of aluminium mask was applied on the PV devices to obtain an active area of 0.04 cm^2^ and to prevent any contribution from externally fallen light on the devices. EQE spectra were measured by a home-built spectral response measurement set-up. X-ray diffraction (XRD) patterns were obtained using Philips X’PERT MPD with operational parameters of 40 kV tube voltage and 40 mA tube current. SEM was used to investigate morphologies of the perovskite thin films by FEI Nova Nano200 Microscopic instrument. SEM samples were all Au-coated to eliminate charge collection. AFM images were obtained by Bruker multimode 8. Steady-state PL spectra were recorded by the Varian cary eclipse fluorescence spectrophotometer upon excitation 400 and 620 nm, separately. Light absorption spectra measurements were carried out by the Varian 50 Scan UV–Vis spectrophotometer. Stability investigation was carried out for devices kept in the glovebox under dark conditions except taking out for testing of J-V performance or at ambient air with ~35% humility. FTIR spectra were recorded in the frequency range of 650–1800 cm^−1^ using Nexus FTIR instruments (Thermo Nicolet Corp, USA) with ATR-FTIR spectrometer. FTIR thin film samples were prepared on the Au-coated glass slides.

## Additional Information

**How to cite this article**: Wang, H. *et al.* A composite light-harvesting layer from photoactive polymer and halide perovskite for planar heterojunction solar cells. *Sci. Rep.* **6**, 29567; doi: 10.1038/srep29567 (2016).

## Supplementary Material

Supplementary Information
