# Supplementary material for: A composite light-harvesting layer from photoactive polymer and halide perovskite for planar heterojunction solar cells
Source: Sci Rep. 2016 Jul 14;6:29567. doi: 10.1038/srep29567 (PMC4944171; doi:10.1038/srep29567)
Supplement: Supplementary Information [file srep29567-s1.pdf]

Supplementary Information

A composite light-harvesting layer from photoactive polymer and halide perovskite for planar heterojunction solar cells

Heming Wang*, Yaqub Rahaq, Vikas Kumar

Materials & Engineering Research Institute, Sheffield Hallam University, City Campus, Howard Street, Sheffield, S1 1WB

* h.wang@shu.ac.uk

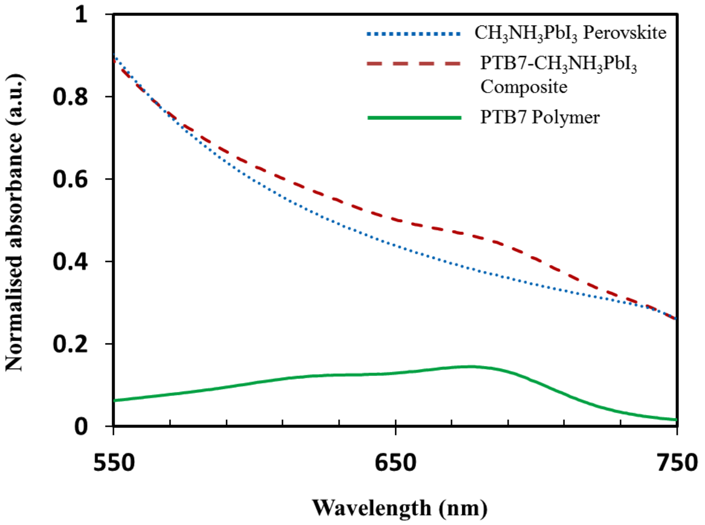

Figure S1 Light absorbance of PTB7 polymer, $CH_3NH_3PbI_3$ perovskite, and PTB7-$CH_3NH_3PbI_3$ composite.

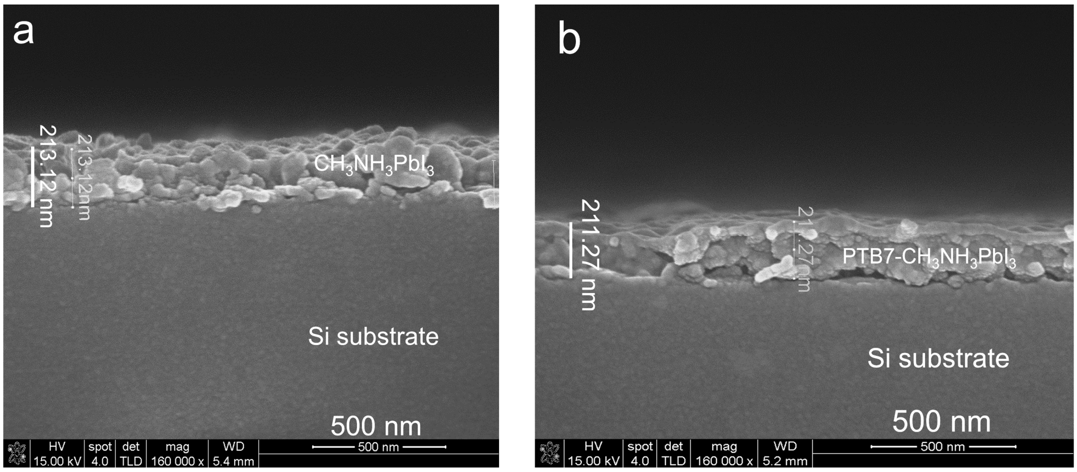

Figure S2 Cross section SEM images of photoactive thin films on the Si substrates, respectively: a) $CH_3NH_3PbI_3$ perovskite; b) PTB7-$CH_3NH_3PbI_3$ composite.

Table S1 Performance of solar cells based on PTB7-$CH_3NH_3PbI_3$ composite

No. of Devices	V_{oc} (V)	J_{sc} (mA/cm²)	PCE (%)	FF
1	0.87	22.6	12.90	0.65
2	0.86	22.4	12.70	0.65
3	0.90	23.7	13.40	0.62
4	0.90	22.8	12.72	0.61
5	0.91	21.8	12.60	0.63
6	0.90	22.8	14.40	0.70
7	0.83	21.4	11.10	0.62
8	0.84	21.1	11.27	0.63
9	0.84	22.0	11.78	0.63
10	0.90	22.2	12.23	0.61
Mean	0.88	22.28	12.51	0.63
Standard Deviation	0.031	0.76	0.99	0.027

Table S2 Performance of solar cells based on $CH_3NH_3PbI_3$ perovskite

No. of Devices	V_{oc} (V)	J_{sc} (mA/cm²)	PCE (%)	FF
1	0.88	20.0	11.59	0.66
2	0.85	18.9	11.50	0.71
3	0.87	22.7	14.02	0.70
4	0.87	22.0	13.60	0.71
5	0.84	21.9	13.09	0.71
6	0.88	23.0	14.00	0.69
7	0.85	20.0	12.07	0.71
8	0.88	19.4	12.10	0.70
9	0.88	23.6	14.30	0.69
10	0.87	19.7	12.42	0.72
11	0.85	19.2	11.80	0.72
12	0.86	20.2	12.72	0.73
Mean	0.86	20.88	12.76	0.70
Standard Deviation	0.015	1.65	1.01	0.019

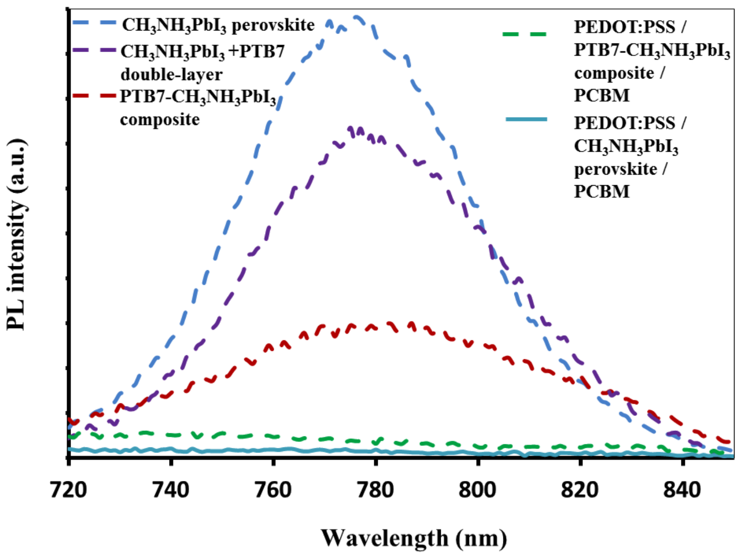

Figure S3 Emission peaks in steady-state photoluminesence spectra upon excitation 620 nm.

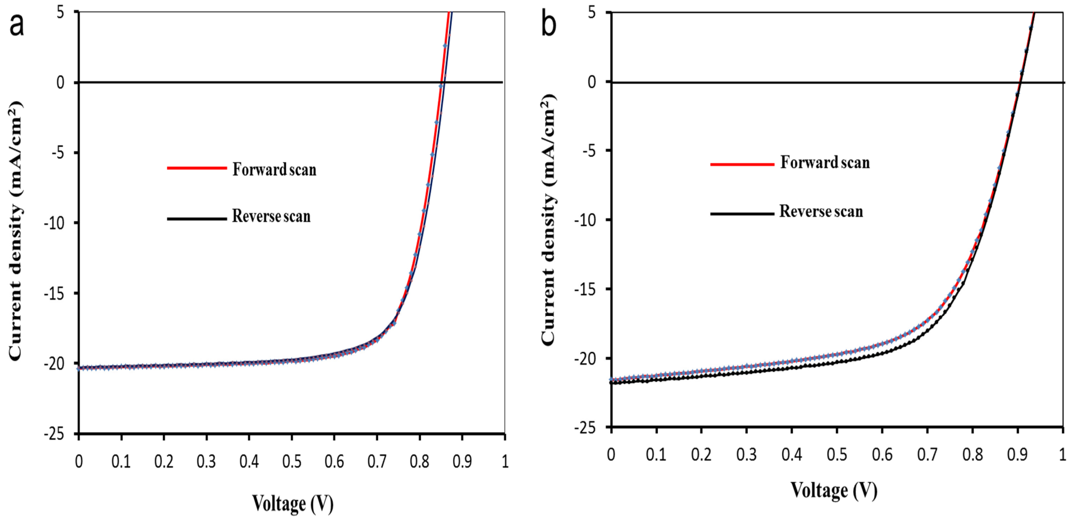

Figure S4 Hysteresis behavior of solar cells: a) J-V curves of $CH_3NH_3PbI_3$-based devices; b) J-V curves of PTB7-$CH_3NH_3PbI_3$-based devices. Both were measured under forward ($J_{sc}\rightarrow V_{oc}$) and reverse ($V_{oc}\rightarrow J_{sc}$) scan at the scan rate of 0.01 V/s.

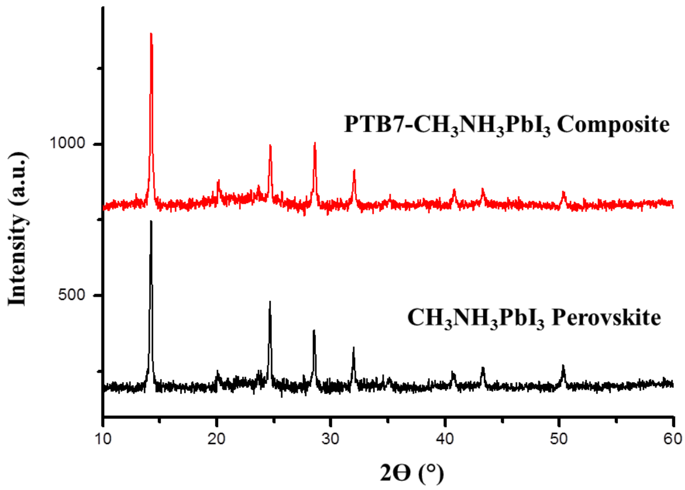

Figure S5 XRD patterns of $CH_3NH_3PbI_3$ perovskite and PTB7-$CH_3NH_3PbI_3$ composite thin films.

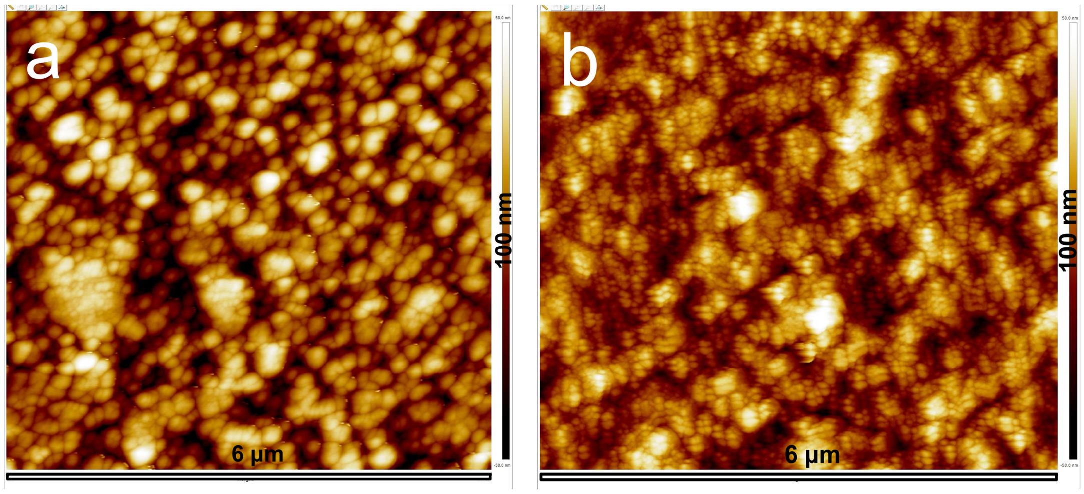

Figure S6 AFM images of photoactive thin films: a) $CH_3NH_3PbI_3$ perovskites; b) PTB7-$CH_3NH_3PbI_3$ composites. Scale bars are 6 x 6 µm and height bars are 100 nm.

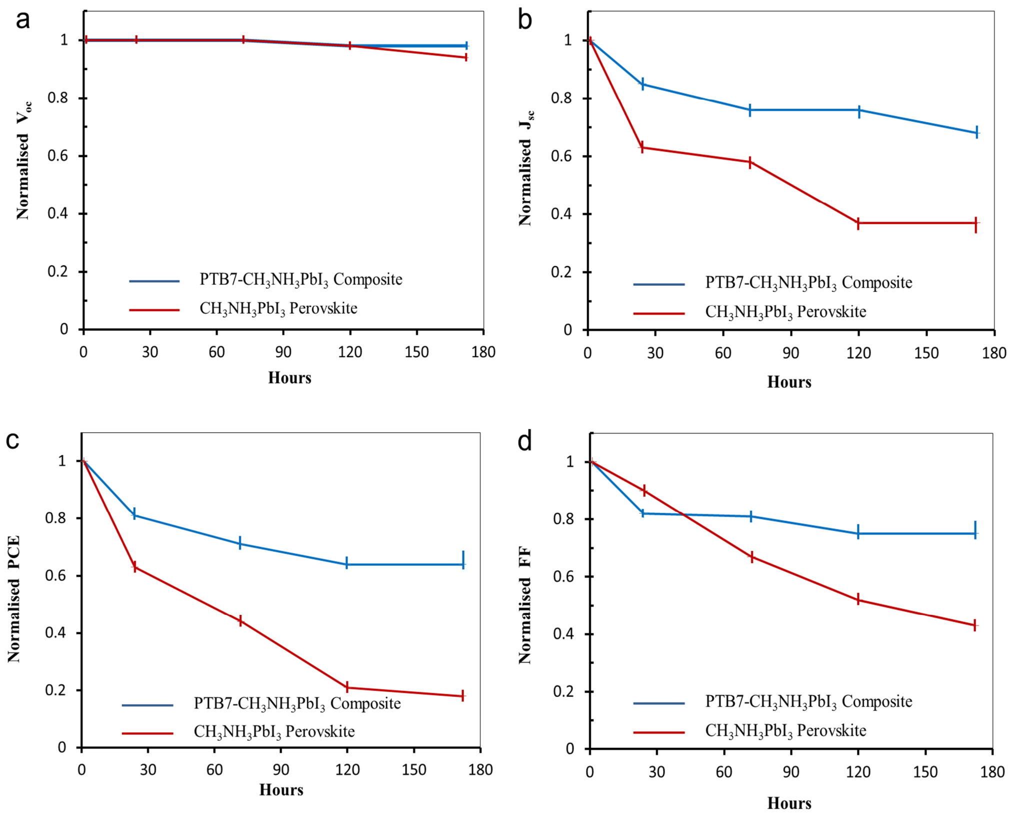

Figure S7 Performance degradation of solar cells under exposure to ambient air against time: a) V_{oc} variation; b) J_{sc} variation; c) PCE variation; d) FF variation.
